# Missed Monteggia Injuries in Children and Adolescents: A Treatment Algorithm

**DOI:** 10.3390/children11040391

**Published:** 2024-03-25

**Authors:** Kristofer Wintges, Christopher Cramer, Konrad Mader

**Affiliations:** 1 Department of Pediatric Surgery, University Medical Center Hamburg-Eppendorf, 20246 Hamburg, Germany; 2 Division Hand, Forearm and Elbow Surgery, Department of Trauma and Orthopaedic Surgery, University Medical Center Hamburg-Eppendorf, 20246 Hamburg, Germany; c.cramer@uke.de

**Keywords:** missed Monteggia injury, radial head dislocation, children, upper extremity deformity, ulnar osteotomy, algorithms

## Abstract

Monteggia injuries are rare childhood injuries. In 25–50% of cases, however, they continue to be overlooked, leading to a chronic Monteggia injury. Initially, the chronic Monteggia injury is only characterized by a moderate motion deficit, which is often masked by compensatory movements. Later, however, there is a progressive valgus deformity, neuropathy of the ulnar nerve and a progressive deformity of the radial head (“mushroom deformity”) with ultimately painful radiocapitellar arthrosis. In the early stages, when the radial head is not yet deformed and there is no osteoarthritis in the humeroradial joint, these injuries can be treated with reconstruction procedures. This can be achieved either by an osteotomy of the proximal ulna with or without gradual lengthening. If there is already a severe deformity of the radial head and painful osteoarthritis, only rescue procedures such as functional radial head resection or radial head resection with or without hemi-interposition arthroplasty can be used to improve mobility and, above all, to eliminate pain. In this review article, we provide an overview of the current treatment options of chronic Monteggia injury in children and adolescents and present a structured treatment algorithm depending on the chronicity and dysplastic changes.

## 1. Introduction

**Figure 1:**
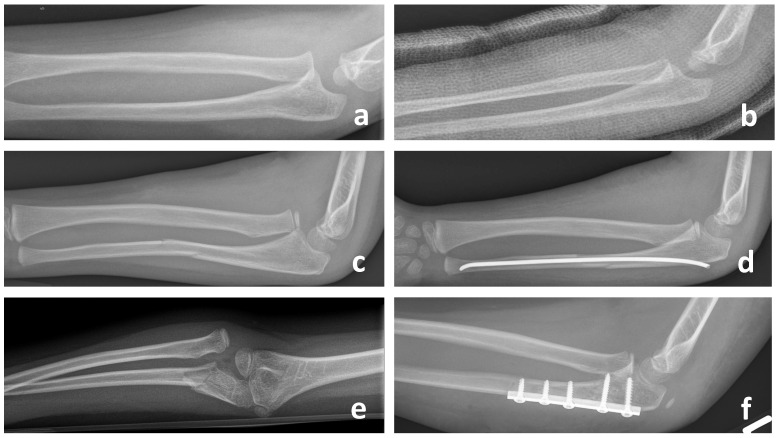
Appropriate identification and treatment of acute Monteggia fracture-dislocations for Bado Typ I (**a**,**c**) and Typ III (**e**) in children with closed reduction and casting (**b**), intramedullary nailing (**d**) or plating (**f**).

Monteggia fracture-dislocations are a rare injury and comprise only approximately 1–2% of all fractures in children [1]. Monteggia first described the missed Monteggia fracture in 1814. It is characterized by a proximal ulnar shaft fracture with a radial head dislocation and is common between the ages of 4 to 10 years. The usual mechanism of injury is a fall onto a pronated and extended arm [2]. Bado classified Monteggia fractures by defining four subtypes depending on the direction of the radial head dislocation and the angulation direction of the ulna fracture [1]. In children and adolescents, Type I injuries (anterior dislocation of the radial head and anteriorly angulated ulnar shaft fracture) or Type III injuries (lateral or anterolateral dislocation of the radial head and metaphyseal ulnar fracture) are most common [3]. With early identification and appropriate treatment with closed reduction and casting, reduction and intramedullary nailing or plating, Monteggia fracture-dislocations show excellent recovery (Figure 1). Unfortunately, this injury is missed in up to 25–50% of the cases due to only subtle signs and symptoms, and only if a greenstick or plastic deformation of the ulna or re-dislocation due to incorrect immobilization is present [4,5]. If the fracture-dislocation persists for more than 4 weeks, it is defined as chronic Monteggia or a missed Monteggia injury [6]. In most of the neglected radial head dislocations, there are no, or only mild, symptoms. Usually, it is diagnosed several months after the trauma when patients complain of moderate pain in the elbow and restricted movement [7]. Due to complex post-traumatic changes, the initial moderate flexion deficit increases due to the excessive growth of the radius compared to the already shortened ulna. A progressive valgus deformity occurs due to the abnormal growth of the radial column with posterolateral instability [8,9]. In addition, the radial head loses its concavity and forms a so-called “mushroom deformity” [10,11]. Similarly, the surfaces of the proximal radioulnar joint (PRUJ) develop abnormally due to the lack of articulation and become incongruent with a loss of concavity on the articular surface and hypertrophy of the humeral capitulum and lateral epicondyle [5,12]. Ultimately, this process ends in painful radiocapitellar osteoarthritis and neuropathy of the ulnar nerve [13]. Hardly any other injury is more dependent on chronicity and the associated anatomical changes. While fracture-dislocations can typically be treated with minimal effort that results in excellent outcomes, Monteggia injuries that have been missed for many years require complex treatment methods. In this review, we aimed to analyze and summarize the treatment options described in the literature for missed Monteggia injuries. We then propose developing a specific treatment algorithm based on the chronicity of the injury and the presence of dysplastic changes.

## 2. Examination and Radiographic Assessment

After obtaining a detailed medical history, which should include restrictions in daily life, as well as the pain and medication profile, hobbies and handedness, the affected arm is clinically examined in comparison to the healthy opposite side. The range of motion is primarily determined using the neutral zero method. The neutral zero method measures all joint movements from a consistently defined zero position. This position corresponds to the anatomical position, which is the standard posture of a healthy person standing upright with their arms at their sides and palms facing forward.

**Figure 2:**
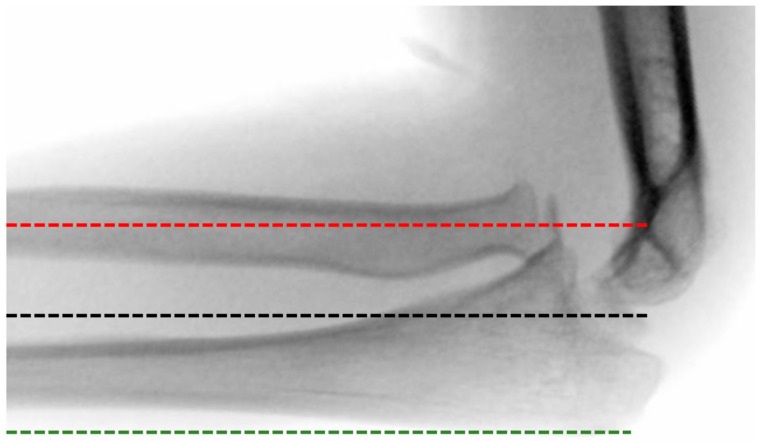
Lateral radiograph of the elbow joint in an 8-year-old girl with a missed Monteggia fracture-dislocation. In preoperative planning, the Støeren line (red line) clearly deviates from the normal radiocapitellar line (black line) and does not intersect the humeral capitulum. Additionally, the ulna already shows plastic deformation, reflected in the ulnar bow sign (green line).

Attention should be paid to the extension and flexion of the elbow and wrist, as well as the rotation of the forearm (pronation/supination). In addition, a cubitus valgus or varus is documented and the arm is examined for additional pain in the wrist and existing neuropathy of the ulnar nerve. Radiographic evaluation of the missed Monteggia injury must include anteroposterior and lateral views of the forearm, including the elbow and wrist. The Støeren line, or radiocapitellar line, is one of the key lines used to assess alignment in the pediatric elbow radiograph. It is a straight line drawn down the neck of the radius and should intersect, or even run through, the center of the humeral capitellum to ensure perfect alignment of the radiocapitellar joint. In chronic Monteggia injuries, however, this line runs off the center of the capitellum humeri (Figure 2), which is a sign of radial head dislocation [14]. In healthy children’s elbows, however, only about 74.5% of the Radiocapitellar Line (RCL) passes the middle third of the capitellum in the apical view, and 93% in the lateral view. Notably, the RCL may entirely miss the humeral capitellum, especially in children under 4 years old. This discrepancy lessens with increasing age, likely due to the presence of a substantial, but still invisible, cartilaginous portion in young children [15]. Therefore, to avoid overdiagnosis in addition to frequent overlooking, direct imaging with ultrasound or MRI should be considered if a misalignment is suspected. Alternatively, X-rays can be examined for the presence of an accompanying “ulnar bow sign”.

An “ulnar bow sign” is verified by a straight line drawn along the dorsal border of the ulna from the olecranon to the distal ulnar metaphysis [16]. A maximum perpendicular distance of more than 1 mm between this line and the ulnar shaft is a clear indication of a plastic deformity of the ulna and should raise the suspicion of a missed Monteggia injury (Figure 2). In addition, a valgus or varus, as well as an ulna-plus variance due to a long-standing dislocation of the radial head and a deformity of the radial head, must be ruled out or taken into account when planning treatment. In the case of complex deformities, a CT scan with a 3D reconstruction of the elbow and proximal forearm should always be performed in order to precisely analyze the position, deformity and arthrosis of the radial head and humerus, as well as any dysmorphia of the PRUJ. In addition, computer tomography can be used to determine the exact height of the radial head resection and 3D corrections can be precisely planned using rapid prototyping technology (RPT) for customized 3D drilling and sawing devices. Depending on the clinical and radiological findings, a structured treatment plan is drawn up with the choice of the appropriate treatment method depending on the patient’s age, the duration of the dislocation and the morphological changes to the radial head and PRUJ. Either anatomical reconstruction procedures with good functional results or, in the case of long-lasting deformity and established osteoarthritis, rescue procedures with good pain reduction and functional improvement are performed.

## 3. Physical Therapy

There is no role for nonoperative management described in the literature for chronic Monteggia injuries. While physical therapy can offer short-term improvement in range of motion, it does not address the progressive deformity with increasing movement restriction and the resulting radiocapitellar osteoarthritis. Therefore, early surgical treatment is the only way to improve mobility after missed Monteggia fractures. Postoperative supportive physical therapy beginning 3–4 weeks after cast removal can help to minimize joint stiffness, improve preoperative muscle atrophy and enhance range of motion.

## 4. Operative Therapy

**Figure 3:**
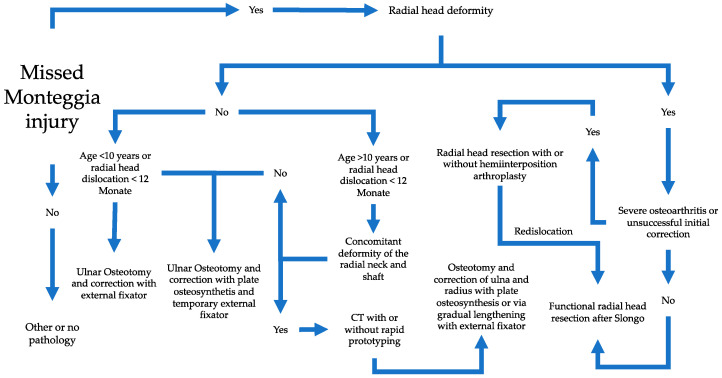
Treatment algorithm based on the chronicity and dysplastic changes (according to the literature).

Treatment is based on the extent of the dysplastic changes and not on the duration of the dislocation as shown in the following treatment algorithm (Figure 3).

Injuries detected early and with no deformation of the radial head and no osteoarthritis in the humeroradial joint can still be treated with reconstruction procedures. The duration between the injury and successful radial head reduction varies in the literature between 12 months and 10 years [7,17,18,19,20]. A good functional result depends on the age of the patient and the remodeling capacity of the radial head, which, according to several studies, is still sufficiently present under the ages of 10 to 12 [20,21]. Overcorrective osteotomy of the ulnar deformity is the most important factor for a safe reduction of the radial head and a good functional outcome. This usually requires angulation with lengthening of the ulna opposite the direction to the dislocation of the radial head. In children under 10 years of age and with a duration of radial head dislocation less than 12 months, proximal ulnar osteotomy and correction with an external fixator or plate osteosynthesis are the most commonly used treatment procedures. 

According to the literature, children who were older than 10 years at the time of surgical treatment or with a duration of radial head dislocation of more than 12 months have a significantly worse functional outcome and a higher dislocation recurrence rate in most cases [22,23,24,25,26]. We therefore always recommend a further diagnostic CT examination for children older than 10 years and with a dislocation of the radial head for more than 12 months to diagnose any concomitant deformations of the radial neck and shaft that may have arisen as a result of the long-term deformity in order to plan a corrective osteotomy of the ulna and radius [7,20,27] or, if necessary, a lengthening osteotomy of the ulna with an external fixator. However, if the dysplastic changes of the radial head and painful osteoarthritis of the radiocapitellar joint have progressed to such an extent that reconstruction procedures can no longer be sensibly carried out, only rescue procedures can be carried out in order to achieve mobility and, above all, a reduction in pain. There are two main surgical options depending on the severity of the osteoarthritis. If severe osteoarthritis is not yet present, a functional resection according to Slongo can be performed. However, if severe osteoarthritis or a previous corrective osteotomy are already present, or functional radial head resection has failed, the last resort is a radius head resection with or without interposition arthroplasty.

### 4.1. Reconstruction Procedures

#### 4.1.1. Ulnar Osteotomy and Correction with External Fixator

**Figure 4:**
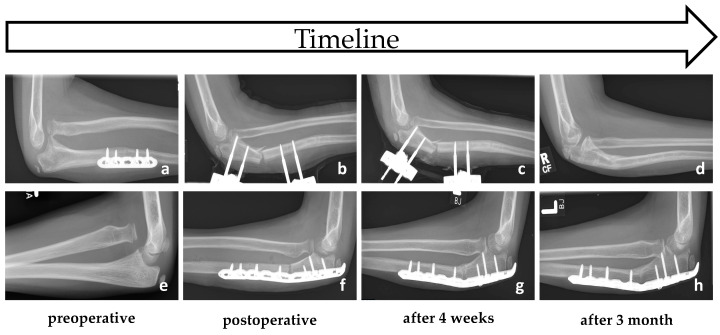
This figure shows the treatment course for the reconstruction of two missed Monteggia fractures in 9-year-old children. Panels (**a**–**d**) depict an angulating proximal ulnar osteotomy with an external fixator and closed reduction of the radial head. This approach was undertaken after a failed external osteotomy at the CORA using plate osteosynthesis. Panels (**e**–**h**) illustrate an angulating proximal ulnar osteotomy with plate osteosynthesis combined with an open reduction of the radial head. In both cases, the far cortex of the osteotomy was left intact to promote faster and improved healing without bone grafting. Radiographs at 4 weeks (**c**,**g**) already demonstrate evidence of consolidation, with complete healing observed at 3 months (**d**,**h**) and no signs of nonunion.

A treatment option for chronic Monteggia injuries under the age of 10 years is a percutaneous subtotal osteotomy of the ulna with closed reduction of the radial head using a miniaturized external fixator (Figure 4a–d) [28,29]. After determining the osteotomy site, two fixator clamps are placed percutaneously proximally and distally under fluoroscopic control and the percutaneous osteotomy of the proximal third of the ulna is performed via an approx. 1 to 2 cm long approach. We recommend an osteotomy of the proximal ulna at least 1 cm distal to the coronoid process, which reinforces the downward pull of the interosseous membrane. This maximizes tension on the interosseous membrane, which contributes to the reduction of the radial head and prevents subluxation or re-dislocation [5,17,22]. In addition, an osteotomy chosen as proximally as possible improves the advantage of a larger bone contact surface, which results in faster and better bone healing and eliminates the need for bone grafting. In children of this age, excellent bone healing typically obviates the need for additional bone grafting. However, it is better to preserve the far cortex of the osteotomy for better and faster healing of the osteotomy gap (Figure 4b–d,f–h). According to the literature, if a wedge gap of more than 1 cm remains at the osteotomy site, a primary bone graft from the iliac crest [30] or an osteoconductive bone graft substitute [31] can be used to prevent nonunion. However, if such a large osteotomy gap occurs, a lengthening of the ulna is more likely to be indicated.

An alternative to proximal ulnar osteotomy is CORA-based osteotomy (Figure 4a). However, it is often difficult to determine the center of rotation and angulation due to the long-standing injury. While some authors theorize that an osteotomy at the CORA restores a normal width of the interosseous membrane and at the same time eliminates the anteriorly bent deformation of the ulna, they believe this is an obstacle for radial head repositioning. Several studies report an increased risk of radial head dislocation and reduced forearm rotation in osteotomies at the CORA, which is why a proximal ulnar osteotomy is preferred in the literature [20,31,32]. After osteotomy of the ulna, the closed reduction of the radial head is enabled by angulation, flexion and lengthening of the ulna. If an anatomical reduction of the radial head is not possible, an open reduction with a resection of the scar tissue is performed via the Kocher approach [28,33]. The fixator remains in place for 6–12 weeks and can be removed on an outpatient basis without anesthetic. 

#### 4.1.2. Ulnar Osteotomy and Correction with Plate Osteosynthesis

**Figure 5:**
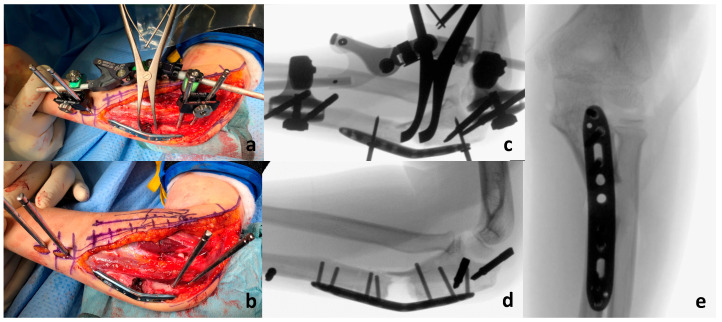
Intraoperative images of the patient: Panels (**a**,**b**): Open reduction of the radial head and osteotomy of the proximal ulna with a temporary external fixator for alignment before definitive osteosynthesis using an advanced locking plate via an extended Kocher approach. Panels (**c**–**e**): Intraoperative fluoroscopy demonstrates correct reduction of the radial head in both planes and the proximal ulnar osteotomy with a remaining dorsal bony bridge. Image c corresponds to image a and image d to the intraoperative image b.

In older children and adolescents and in cases where the use of an external fixator is not an option due to lack of compliance or if the patient and/or parents do not prefer, a corrective proximal ulnar osteotomy and stable plate osteosynthesis is recommended. A subtotal dorsal osteotomy of the proximal third of the ulna is performed by an extended Kocher approach through an additional dorsal window in the anconeus muscle, whereby the length and angulation of the ulna is adjusted under radiological control until a reduction of the radial head is achieved. Another osteotomy option for the lengthening of the ulna without bending is a Z-shaped stepped incision osteotomy [34]. However, a lower dislocation recurrence rate and better functional outcome can be achieved with angulation through the lengthening of the ulna in the direction opposite to the dislocation of the radial head [17,22]. In most cases, open reduction of the radial head is required as the remnants of the original ligament and scar tissue impacts into the joint. After reduction of the radial head and temporary fixation with a monolateral external fixator, a pre-bent plate osteosynthesis is used (Figure 5a–e) [35]. Instead of an external fixator, K-wire fixation through the capitellum into the radial head may be used temporarily to maintain the reduction of the radial head. In rare cases with an irreducible radial head, an additional corrective osteotomy of the proximal radius may be required [36]. In these complex cases, computer-aided 3D planning with RPT technology is recommended [37,38]. This procedure can also be used to correct an existing valgus or varus deformity by flexing the ulna. The authors recommend immobilization in a plaster cast 2 weeks after surgery, followed by a motion orthosis (ROM: Ex/Flex: 0–20–120°) for a further 4 weeks. Hardware removal is recommended after 6–12 months, depending on bone consolidation.

#### 4.1.3. Ulnar Osteotomy and Correction via Gradual Lengthening with External Fixator

If a length discrepancy is present in addition to the radial head dislocation due to a long-standing Monteggia injury, a complex corrective procedure is required. Multiplanar correction of the deformity can be performed either with a monolateral fixator or in complex deformities with a computerized ring fixator [39,40,41,42]. Gradual distraction should start after a latency of 5–7 days at a rate of 3 × 0.25 mm to a maximum of 4 × 0.25 mm per day, and continued until reduction of the radial head is achieved. Once consolidation of the distraction callus is complete, the fixator is removed. Although it was difficult to achieve in a quite large series, Loose et al. were able to show that a concentric reduction of the dialing head could be maintained in an intermediate long-term follow-up [43]. Alternatively, plate osteosynthesis can be used to stabilize the bone regeneration to prevent delayed or nonunion. 

#### 4.1.4. Annular Ligament Reconstruction (ALR)

Whether a reconstruction or repair of the annular ligament should be performed is controversially discussed in the literature. Various reconstruction procedures for ALR using the triceps fascia or palmaris longus tendon, or by direct suture of remnants of the original ligament, have been described [22,32,44]. Some authors are concerned about a lack of stability of the radial head and insist on ALR [30,45]. However, other authors reported no significant benefit of ALR in the treatment of missed Monteggia injuries, as open reduction and lengthening with angulation of the ulna is often sufficient to ensure stability of the radial head by loading the interosseous membrane without ALR or transarticular radiocapitellar pinning [26,31,36,46]. In addition, ALR can lead to osteolytic changes, heterotopic ossification and even avascular osteonecrosis of the radial head, narrowing or growth disturbance of the radial neck, radioulnar synostosis and loss of pronation [18,45,47,48], which is why the authors do not recommend performing this procedure.

### 4.2. Rescue Procedures

#### 4.2.1. Functional Radial Head Resection According to Slongo

**Figure 6:**
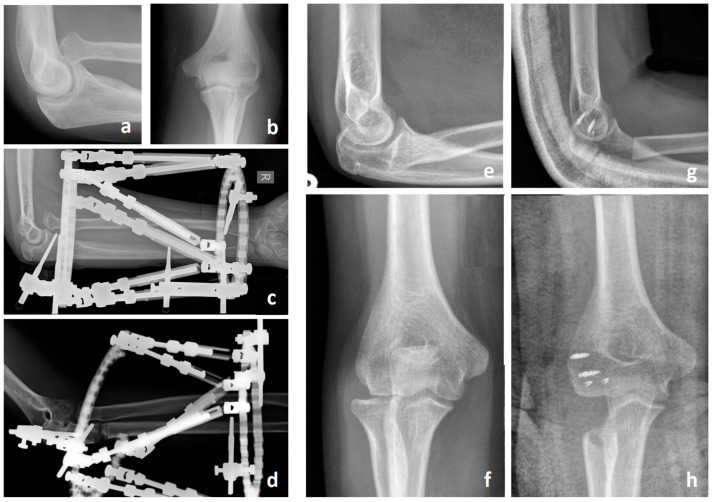
This figure demonstrates two salvage procedures for patients with advanced dysplastic changes of the radial head and proximal radioulnar joint (PRUJ) (**a**,**b**,**e**,**f**). Panels (**a**–**d**) depict a functional radial head resection according to Slongo. This procedure involves distalization of the radial head beyond the capitellum using a ring fixator and a proximal ulnar osteotomy (**c**,**d**). Panels (**e**–**h**) illustrate a radial head resection with hemi-interposition arthroplasty. In this approach, the radial head is resected distal to the coronoid process. A capsular flap is interposed between the humeral capitulum and the resected radial head to act as a spacer with small suture anchors. Additionally, the detached lateral ulnar collateral ligament is reattached to the bone with suture anchors.

One possible treatment option is the “functional radial head resection” according to Slongo, using a computerised external ring fixator. This technique is a gradual angulation and distalization technique in which the ulna and radial head are lengthened and simultaneously distalized using a half-ring fixator or ring fixator [43,49,50]. However, the aim of this operation is not to restore a functional joint, but merely to distalize the radial head. The usual distalization length of 1–2 cm beyond the capitellum to a more distal position enables a “functional” resection and creates a buffer zone in the dysplastic joint (Figure 6). Transfixation of the radius must be ensured during the entire consolidation phase to prevent secondary dissociation in the DRUJ. Gradual correction is achieved by lengthening the ulna by 0.5 to 1.0 mm per day and angulation depending on the post-traumatic deformity with a delay of 5–7 days after the procedure. Lengthening is computerized, e.g., via an app, and is monitored daily by the patient for 3 months. As soon as the ulna osteotomy has consolidated, the external fixator can be removed. If union is delayed, a plate osteosynthesis or a plaster cast is applied for a further 2 to 4 weeks. Delayed or nonunion, pin infection, re-dislocation of the radial head and an ulnar-plus variance have been described in the literature as complications of this procedure (Table 1) [33,43,49].

This table summarizes the complication rates in the literature for the rescue procedure of functional radial head resection according to Slongo and radial head resection. The individual complication rates are given as a fraction of the total number of patients.

#### 4.2.2. Radial Head Resection with or without Hemi-Interposition Arthroplasty

In the event of a failed corrective osteotomy or distalization with re-dislocation of the radial head or severe osteoarthritis, radial head resection with or without hemi-interposition arthroplasty is the last rescue procedure to be considered. For this rescue procedure, elbow instability and an ulna-plus variance has to be ruled out, as otherwise impingement of the lateral ulnar collateral ligament [57], rotational instability [58], limitation of movement [59] or subsequent osteoarthritis caused by increased load on the coronoid [60] may occur. In addition, proximal migration of the radius, resulting in wrist pain and restricted mobility of the elbow and wrist, as in the case of an Essex–Lopresti lesion, can occur [61,62]. The plastically deformed radial head is resected at the radial head–neck junction by an extended Kocher approach. To reduce possible radiocapitellar osteoarthritis and proximal migration of the radius, the extended ventral capsule is sutured over the capitulum [12,52] or an anconeus muscle interposition [53], an Achilles allograft interposition graft [55] or a corium graft [63] to create an “unconnected spacer” for the proximal radius as a hemi-interposition arthroplasty is used. Over time, the resected proximal radius forms a “neoradial head”, but without full function and force absorption. If the lateral ulnar collateral ligament (LUCL) has been detached, it is refixed with a suture anchor system to create lateral stability. The authors recommend immobilization in a plaster cast 2 weeks after surgery, followed by a motion orthosis (ROM: Ex/Flex: 0–20–120°) for a further 4 weeks. Due to the occurrence of proximal migration of the radius (3.4–5.4 mm after 7–10 years) [52,54], perifocal ossification and recurrent synostosis [52], reformation of the radial head [64], as well as cubitus valgus and instability [51,53,56], repeated revision surgery may be required, which is why radial head excision is only recommended in the pediatric population shortly before or after skeletal maturity (Table 1).

## 5. Conclusions

The treatment of a chronic Monteggia fracture-dislocation is a significant challenge in orthopedics, because it is often overlooked at initial medical care, possibly due to limited knowledge of the pediatric elbow anatomy or only subtle initial clinical symptoms. Diagnosing a chronic Monteggia injury necessitates a good treatment concept for the frequently complex deformity, as anatomical changes to the elbow can occur within 3 months. Depending on the morphological changes of the radial head and the PRUJ, either anatomic reconstruction procedures with good functional results or, in the case of long-lasting deformity and existing osteoarthritis, rescue operations with good pain relief and functional improvement are used. For this purpose, treatment is only performed in centers with many years of experience that surgically treat at least 5 to 10 cases per year. Importantly, preventing Monteggia fracture-dislocation remains the primary objective, as treatment of acute injuries offers the most favorable outcomes.

## Figures and Tables

**Table 1:** Complication rates after rescue procedures.

	Functional Radial Head Resection	Radial Head Resection
Delayed or Nonunion	1/16 [43], 2/15 [33]	-
Pin Infection	2/15 [33]	-
Re-dislocation	0 [33,43,49]	-
Ulnar positive variance	2/16 [43]	3/5 [51], 12/27 [52], 1/7 [53], 4/16 [54], 3/4 [55],
Re-operation	1/16 [43], 2/15 [33]	2/5 [51], 4/16 [54], 1/4 [55], 7/27 [52], 7/27 [52]
Cubitus valgus	-	1/5 [51], 3/7 [53],1/2 [56]
Instability	-	1/2 [56]

## Data Availability

Not applicable.
